# A High-Performance System for Robust Stain Normalization of Whole-Slide Images in Histopathology

**DOI:** 10.3389/fmed.2019.00193

**Published:** 2019-09-30

**Authors:** Andreea Anghel, Milos Stanisavljevic, Sonali Andani, Nikolaos Papandreou, Jan Hendrick Rüschoff, Peter Wild, Maria Gabrani, Haralampos Pozidis

**Affiliations:** ^1^IBM Research – Zurich, Rüschlikon, Switzerland; ^2^Institute of Pathology and Molecular Pathology, University Hospital Zürich, Zurich, Switzerland; ^3^Dr. Senckenberg Institute of Pathology, University Hospital Frankfurt, Frankfurt, Germany

**Keywords:** stain normalization, whole-slide image analysis, large-scale image analysis, tumor detection, convolutional neural networks, digital pathology

## Abstract

Stain normalization is an important processing task for computer-aided diagnosis (CAD) systems in modern digital pathology. This task reduces the color and intensity variations present in stained images from different laboratories. Consequently, stain normalization typically increases the prediction accuracy of CAD systems. However, there are computational challenges that this normalization step must overcome, especially for real-time applications: the memory and run-time bottlenecks associated with the processing of images in high resolution, e.g., 40X. Moreover, stain normalization can be sensitive to the quality of the input images, e.g., when they contain stain spots or dirt. In this case, the algorithm may fail to accurately estimate the stain vectors. We present a high-performance system for stain normalization using a state-of-the-art unsupervised method based on stain-vector estimation. Using a highly-optimized normalization engine, our architecture enables high-speed and large-scale processing of high-resolution whole-slide images. This optimized engine integrates an automated thresholding technique to determine the useful pixels and uses a novel pixel-sampling method that significantly reduces the processing time of the normalization algorithm. We demonstrate the performance of our architecture using measurements from images of different sizes and scanner formats that belong to four different datasets. The results show that our optimizations achieve up to 58x speedup compared to a baseline implementation. We also prove the scalability of our system by showing that the processing time scales almost linearly with the amount of tissue pixels present in the image. Furthermore, we show that the output of the normalization algorithm can be adversely affected when the input images include artifacts. To address this issue, we enhance the stain normalization pipeline by introducing a parameter cross-checking technique that automatically detects the distortion of the algorithm's critical parameters. To assess the robustness of the proposed method we employ a machine learning (ML) pipeline that classifies images for detection of prostate cancer. The results show that the enhanced normalization algorithm increases the classification accuracy of the ML pipeline in the presence of poor-quality input images. For an exemplary ML pipeline, our new method increases the accuracy on an unseen dataset from 0.79 to 0.87.

## 1. Introduction

With the advent of high-resolution whole-slide imaging technology and the advances in deep learning, computer-aided diagnosis (CAD) systems have become a very important part of the clinical work today. Machine learning (ML) based image analysis algorithms applied to digitized histological slides can assist the pathologists in terms of workload reduction, efficient decision support, and interpretability of the results (1–3). Given the vast amount of gigapixel-sized whole-slide imaging data, and the need to accelerate the time-to-insight, there is an increasing demand to build automated and scalable pipelines for large-scale, fast, and robust image analysis.

One of the main pre-processing algorithms in whole-slide image (WSI) analysis is the color normalization of stained tissue samples (4). Despite the standardized staining protocols, variations in the staining results are still frequent due to differences in, e.g., the antigen concentration and incubation time and temperature, the different conditions across slide scanners etc. (3). Such color/intensity variations can adversely affect the performance and accuracy of the CAD systems. Stain normalization methods aim to help the CAD systems by generating images with a standardized appearance of the different stains (5–12).

In this work, we use the Macenko method (7) to build a high-performance stain normalization system. This method estimates the stain vectors of the WSI of interest by using a singular value decomposition (SVD) approach applied to the non-background pixels of the input image. Using the normalized median intensity (NMI) metric, it was shown in Zanjani et al. (13), that the quality of this method is one of the highest when compared to other stain normalization methods. In addition, due to the simplicity of the algorithmic steps, the particular method can be efficiently parallelized. Moreover, the algorithm does not involve intermediate steps that require training of model parameters and is thus computationally less expensive.

Our stain normalization system architecture is based on an optimized multi-core implementation that integrates multiple system-level optimizations (14)^1^. With this architecture we address two challenges of the stain normalization algorithm: (1) long processing time, and (2) large system memory consumption, when normalizing high-resolution images. Typical implementations of stain normalization algorithms cannot process high-resolution images, such as 40X resolution of a 160 k × 80 k WSI corresponding to 37.5 GB of data, on typical servers with less than 64 GB of RAM. Our implementation enables the processing of such images and can be used with different image formats, such as .svs, .tif, .ndpi, etc. This allows us to evaluate the performance of our stain normalization system on datasets generated by different scanners, e.g., Ventana, Hamamatsu, Aperio, Philips.

**Figure 1:**
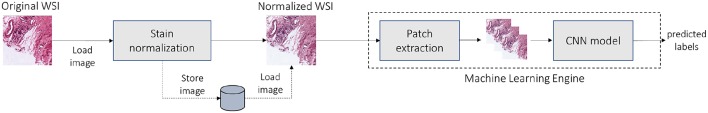
Exemplary ML-based pipeline: stain-normalized WSIs feed an ML engine for tumor detection.

Furthermore, we show that the stain normalization algorithm under study is sensitive to the quality of the input images. To overcome this challenge, we propose a new method to detect the poor-quality images and design a variant of the algorithm that is robust to such images. For the rest of the paper, we will refer to an image as having poor quality, when that image contains artifacts, e.g., stain spots, dirt etc. Finally, we show that our new normalization method can increase the accuracy of machine learning (ML) pipelines that use stain-normalized images as input. As an exemplary ML pipeline, we used a Tensorflow-based convolutional neural network (CNN) that detects tumor in prostate biopsy WSIs. Figure 1 shows a top-level overview of the ML-based pipeline used in this paper. Before feeding the ML engine with histological WSIs, we normalize the full image in a user pre-defined resolution, e.g., 10X. The normalized images can be stored to disk or directly pipelined with the ML engine. Then, the latter trains a CNN model using patches from the normalized WSI. The trained model is then used for inference to predict the presence of tumor in images.

This paper is structured as follows. In section 2 we describe the datasets used in our study. In section 3.1 we describe the stain normalization algorithm employed in this study and re-emphasize the motivation of this work. In section 3.2 we present a high-performance version of the Macenko normalization algorithm that not only speeds up the execution time, but also enables the normalization of large whole-slide images, e.g., in 40X resolution, on off-the-shelf servers with less than 64 GB of RAM. In section 3.3, we show how sensitive the normalization algorithm under study can be to input images of poor quality, e.g., containing artifacts, spots of ink, dirt etc. We continue the section with depicting a method that detects such images and normalizes them using an algorithm variant which is robust to such input. Next, in section 3.4, we present the CNN architecture used to train models for detection of tumor in prostate biopsy whole-slide images. We summarize and discuss the results of the paper in sections 4 and 5, respectively. Finally, we conclude the paper in section 6.

## 2. Materials

### 2.1. Whole-Slide Image Datasets

In this work, we use four datasets that contain H&E-stained whole-slide images, all including 10X and 40X resolutions. Two datasets are publicly available and two are proprietary.

- The first dataset is part of TUPAC MICCAI 2016 (15) and provides breast WSIs for prediction of tumor and proliferation scores. These WSIs are in Aperio format, single-file pyramidal tiled TIFF (.svs), with JPEG compression scheme.
- The second one is the CAMELYON16 dataset (16), which is part of the ISBI challenge on cancer metastasis detection in lymph node. These slides are in Philips format, single-file pyramidal tiled TIFF or BigTIFF (.tif) with non-standard metadata and JPEG compression scheme.
- The third dataset is proprietary. The slides are in Ventana format, single-file pyramidal tiled BigTIFF with non-standard metadata. It contains 96 needle-based biopsy images of prostate cancer tissue. These images were digitized with the Ventana scanner providing a resolution of 0.25 microns.
- The fourth dataset is also proprietary and contains whole-slide images in Hamamatsu format, single-file TIFF-like format (.ndpi) with proprietary metadata. This dataset consists of radical prostatectomy tissue images taken from 30 patients. These whole-slide images were generated with the Hamamatsu scanner at a resolution of 0.23 microns.

We use all datasets to analyze the performance, i.e., the run-time, speedup, and scalability, of our stain normalization system. We show that our implementation not only accelerates the normalization pre-processing, but it also supports different whole-slide image formats. In particular, the .ndpi/Hamamatsu format is not yet supported by standard open-source software for large-scale image analysis, such as the OpenSlide library (17). We included in our performance analysis the latter dataset to show the flexibility of our normalization system to load and process different image formats.

To evaluate the impact of stain normalization on the accuracy of ML-based medical pipelines and to assess the robustness of our enhanced normalization method to poor-quality images, we used only one of the proprietary datasets. More specifically, we used the prostate biopsy dataset, as it contains poor-quality images, which is not the case for the publicly available datasets. Thus, we can train and test a neural network model using a reasonably large number of such images. This dataset is the main focus regarding the new robust method presented in section 3.3, because it allowed us to identify shortcomings of the stain normalization algorithm and motivated us to design an enhanced normalization method which is robust to poor-quality images.

The prostate biopsy dataset contains 96 WSIs with tumor regions annotated by two pathologists. The annotations include Gleason scores: non-tumor, 3+3, 3+4, 4+3, 4+4, and 4+5. Modeling this problem as a multi-class classification is not appropriate in our case for several reasons. The distribution of samples across the Gleason scores is imbalanced. Although there are ways to compensate for class imbalance, in this particular case, some classes have so few examples that training on them would not be meaningful. Moreover, at Gleason score granularity, the class patterns overlap heavily, and it is hard to discriminate between classes, e.g., 3+3 and 3+4 or 4+3 and 4+4. Modeling the problem as a binary classification task mitigates these issues and still provides a useful categorization between healthy and tumorous samples. The regions with a Gleason score higher than or equal to 3+3 are considered as tumor and the remaining as non-tumor. Modeling this problem as a binary classification task has also been widely used by the scientific community (18, 19).

## 3. Methods

### 3.1. Stain Normalization of Whole-Slide Images

The stain normalization (SN) method presented in Macenko et al. (7) belongs to the class of unsupervised normalization methods. The algorithm first estimates the hematoxylin and eosin (H&E) stain vectors of the WSI of interest by using a singular value decomposition (SVD) approach applied to the non-background pixels of the input image. Second, the algorithm applies a correction to account for the intensity variations due to the original strength of the stain, staining procedure etc. Finally, the image is projected to a reference image such that after stain normalization all normalized images have similar color characteristics. The algorithm is based on the principle that the color of each pixel (RGB channels) is a linear combination of the two H&E stain vectors which are unknown and need to be estimated. A reference MATLAB implementation of the Macenko algorithm is publicly available in (20). We outline the algorithmic steps of the Macenko normalization method in Algorithm 1. Most of the processing steps are applied to the RGB color vectors converted to optical density (OD) domain. Each RGB vector *I* with the color components normalized to [0,1] is transformed as follows *OD* = −log_10_(*I*). This transformation provides a space where a linear combination of stains results in a linear combination of OD values.

**Algorithm 1:** The Macenko stain normalization algorithm.

1: Convert RGB to optical density (OD)
2: Remove pixels with negligible optical density
3: Apply SVD on the OD tuples and use the largest 2 values to create the SVD plane
4: Project data onto the plane and normalize to unit length
5: Calculate the angle ϕ of each point with respect to the 1^st^ (or 2^nd^) SVD direction
6: Find the robust extremes (α^*th*^ and (100−α)^*th*^ percentiles) of the angle ϕ
7: Find the projection of the extreme values back to OD space
8: Use this projection as optical density matrix (ODM)
9: Calculate the individual stain concentrations (*C*_*h*_ and *C*_*e*_) using the inverse of ODM
10: Find the robust maximum ((100 − α)^*th*^ percentiles) of the individual stain concentrations *C*_*h*_, *C*_*e*_
11: Normalize and transform concentrations to OD space and then back to RGB using an H&E template

**Figure 2:**
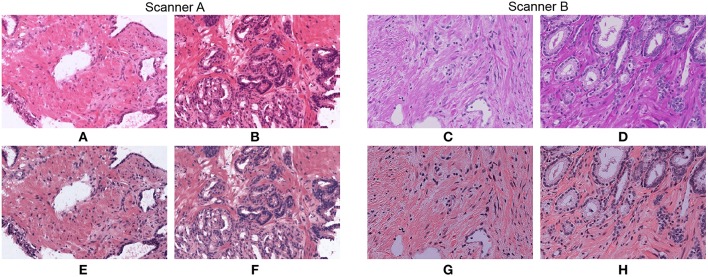
Original patches **(A–D)** and their normalized versions **(E–H)** after applying our optimized stain normalization method fast_sn described in section 3.2.

In Figure 2 we show how stain normalization reduces the stain variability across images within and across different datasets. As a reference image, we have used the H&E vectors and maximum concentration values reported as a template in (20). We use the same reference image for the rest of the paper. To generate these normalized images we have used an optimized implementation of Algorithm 1, that we will describe in detail in section 3.2. We show original and normalized patches extracted from WSIs that belong to the two proprietary datasets described in section 2 and generated by different scanners A and B.

Previous state-of-the-art studies such as Ciompi et al. (21) demonstrated that the classification accuracy of a machine learning-based histopathology system is improved when using stain-normalized images. Other machine learning-based systems that use stain-normalized histopathology images have been proposed in the past. Zerhouni et al. (4) introduce an ML architecture for mitosis detection in breast histopathology WSIs. The approach uses stain-normalized patches of the original image in 40X resolution to train a Wide Residual Network. Ciresan et al. (22) propose an ML-based pipeline also for mitosis detection in breast histopathology images. The study uses H&E-stained WSIs split into patches that feed the training engine of an 11-layer CNN model. Litjens et al. (19) describe an ML pipeline for the detection of prostate cancer in H&E-stained whole-slide biopsy specimens. In general, stain normalization helps significantly to reduce the variability between whole-slide images, especially when they come from different hospitals or laboratories. When the variability between images is small, e.g., when they belong to the same dataset from the same clinic, stain normalization may have small impact on the ML pipeline, as was also shown in Lafarge et al. (23).

**Figure 3:**
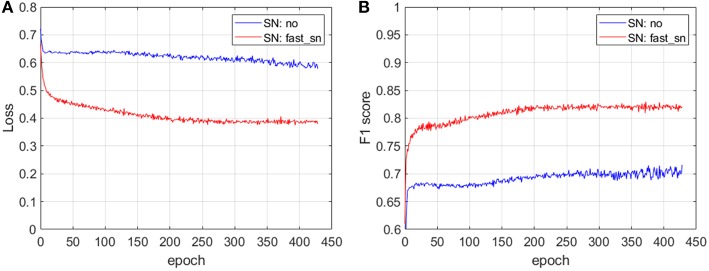
The impact of stain normalization on the accuracy of an ML pipeline. After 430 epochs, training on stain-normalized images leads to a 35% lower validation loss **(A)** and an improvement in F1 score of 11 percentage points **(B)**. The CNN architecture used for these experiments is described in section 3.4.

In Figure 3, we show the prediction accuracy of an ML pipeline with and without applying stain normalization to the input images. For these experiments we have used images from one of the proprietary datasets described in section 2 where the images show variability. We train a convolutional neural network (CNN) architecture with non-normalized images (SN: no) and with normalized images (SN: fast_sn) and we plot the loss (Figure 3A) and F1 score (Figure 3B) on a validation dataset at different training epochs. We describe the CNN architecture in detail in section 3.4. The results depicted in Figure 3 show that after the same number of epochs, i.e., 430, training on stain-normalized images leads to a 35% lower validation loss and to an improvement in F1 score of 11 percentage points. We have also evaluated the generalization strength of the two trained CNN models on an unseen (test) dataset: normalizing the input whole-slide images improves the F1 score by 5 percentage points, from 0.74 (without normalization) to 0.79 (with normalization).

### 3.2. Optimized Stain Normalization of Whole-Slide Images (fast_sn)

An example of a reference MATLAB implementation of Algorithm 1 is publicly available in (20). A direct porting of this implementation to C++ suffers from memory and run-time bottlenecks, especially when trying to process 40X images. For example, the implementation fails to run on systems with 64 GB of RAM on 40X WSIs. Even when increasing the RAM capacity, it suffers from long processing time (>16 min per image on an 8-core Intel(R) Xeon(R) CPU E5-2630 v3) for 40X images. The reason behind these issues is the lack of a system-aware implementation that uses the multi-core processors and the memory of the running system in an efficient way to allow fast loading and processing of the WSIs. For the rest of the paper, we will denote this C++ reference implementation by reference. In this section we present the steps we have taken to build an optimized stain normalization implementation that addresses the drawbacks of the reference implementation. We will refer to this optimized Macenko method implementation as fast_sn.

**Figure 4:**
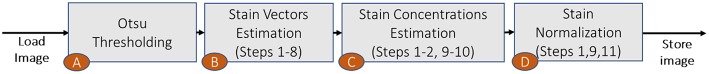
Mapping the steps of Algorithm 1 to our optimized implementation fast_sn.

Before describing the run-time optimizations, we present an algorithm enhancement that we have introduced in our optimized stain normalization implementation (the processing block *A* in Figure 4) in addition to what we have previously presented in Stanisavljevic et al. (14). This first processing step automatically calculates the threshold based on which the pixels with low optical density are removed in Step 2 of Algorithm 1. In the original Macenko method, these pixels are removed based on an empirically-defined value which is common for all WSIs in a dataset. However, due to the intra-dataset color and intensity variability, a unique threshold is usually not recommended, as for some images the algorithm might remove too few or too many pixels and the robust extremes from Steps 6 and 11 in Algorithm 1 could be wrongly identified.

We propose to use an Otsu-based algorithm (24) to automatically identify the correct threshold for each individual image. To reduce the processing overhead, the block *A* in Figure 4 reads the input image in a low resolution, e.g., 2.5X, converts the pixels to gray-scale and applies Gaussian blur to further separate the maximum of the image intensity histograms. The optimal threshold is then extracted using Otsu thresholding. Both the Gaussian blur and the Otsu thresholding functions are integrated from the C/C++ opencv library.

**Figure 5:**
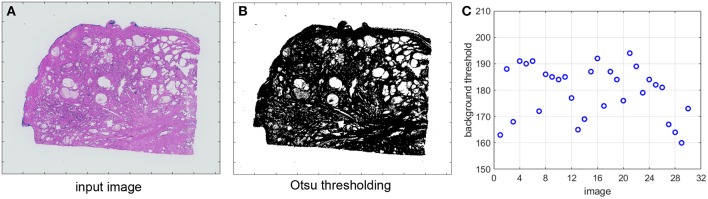
**(A)** Original image. **(B)** Otsu thresholding. **(C)** Otsu-detected thresholds for a set of images.

Figure 5A shows an original image from one of the datasets described in section 2. In Figure 5B we display its corresponding image after applying Otsu thresholding. We further extract the Otsu-detected thresholds for a set of images from the same dataset and we plot them in Figure 5C. The background threshold value is a number between 0 and 255. As we can see in Figure 5C, even though the images belong to the same dataset they can have very different thresholds for filtering the pixels with low optical density. This parameter may therefore significantly affect the output of the SN algorithm. Our proposed automated detection step in Figure 4 ensures the determination of an accurate parameter value for each image.

Next, we present a set of implementation optimizations to decrease the run-time and memory footprint of the Macenko algorithm. The processing blocks *B, C*, and *D* in Figure 4 correspond to the optical density matrix (ODM (5)) calculation, the computation of stain concentrations and their robust maximum values, and the pixel normalization after transformation back to RGB space, respectively. Our implementation follows the steps shown in Algorithm 1. Due to multiple optimizations, we reorganize the steps of the original Macenko algorithm as shown in Figure 4.

(a) After image loading, only the RGB pixel values are stored in CPU memory. Since stain normalization is typically performed in the optical density (OD) domain, the conversion from RGB to OD in the various processing blocks *B, C*, and *D* is performed on the non-background pixels only. A look-up table method is used for the conversion from RGB to OD space instead of the log function in order to speed up the logarithmic computation.

(b) In block *B*, the covariance matrix in Step 3 of Algorithm 1 is calculated using the property that the element (*i, j*) of the matrix, $\Sigma_{ij}=\frac{1}{N^{2}}\left(\underset{p}{\sum }x_{p,i}x_{p,j}-\underset{p}{\sum }x_{p,i}\underset{p}{\sum }x_{p,j}\right)$, requires only the sums of OD components.

(c) In blocks *B* and *C*, which are benchmarked as the most time-consuming steps, we use partial sorting to find the percentiles from Steps 6 and 10 in Algorithm 1. This partial sorting runs 3-4x faster compared to full sorting for our data.

(d) For the exponential function in processing block *D*, we use the fast exponentiation library (25) since it performs 5-10x faster compared to the corresponding function in the standard C library.

(e) Since processing blocks *B-D* perform many independent operations on individual pixels, their execution is parallelized across all available CPU threads using the OpenMP library (26).

(f) Given that processing blocks *B* and *C* are the most time-consuming due to the inherent difficulty of parallelizing the sorting operations, we propose a further optimization that is based on a Monte Carlo sampling technique (27). In this method, a sample of non-background pixels is randomly chosen from the set of all non-background pixels in order to estimate the required robust extremes from Steps 6 and 10. Even though there are different methods for estimating the population percentiles (28), an analytical estimation of the required sample size is difficult (29). Therefore, we derive the optimal sample size based on empirical experimental results. A good sampling rate that we use in this paper is 1%. This rate was found by computing the Euclidean distance of the OD matrix and the relative error of the robust maximum of the individual stain concentrations (max *C*_*h*_ and max *C*_*e*_) between the sampling (with different sampling rates) and no-sampling results (14).

### 3.3. Normalization Method Robust to Poor-Quality WSIs (fast_rsn and fast_rsn_all)

In the previous section, we have shown how to deal with the run-time and memory performance challenges of the Macenko algorithm. In this section we address yet another challenge of this normalization method. We show that the Macenko algorithm may fail to estimate the correct H&E vectors when the quality of the input image is poor, e.g., the image contains artifacts and/or impurities, such as staining spots, dirt, etc. In such cases, the estimated H&E vectors may not be accurate and may be biased toward a specific color (e.g., blue or pink). Such normalization results may adversely impact the accuracy of a machine learning pipeline that uses as input normalized whole-slide images. We demonstrate examples of such cases in section 4.

**Figure 6:**
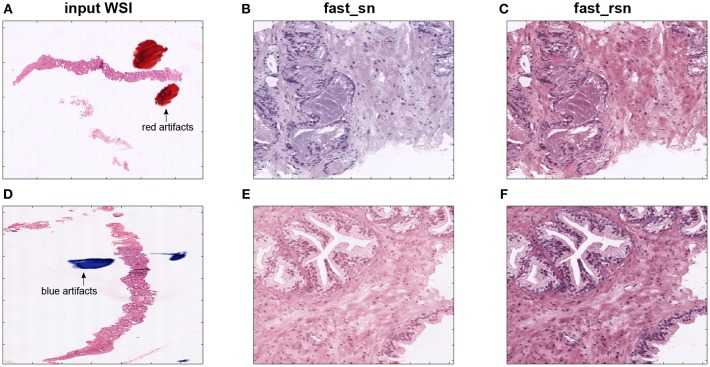
First column **(A,D)** shows two original images from a prostate biopsy dataset. The second column **(B,E)** shows patches extracted from the original images, normalized using fast_sn. The third column **(C,F)** shows the same patches but normalized using fast_rsn.

Figures 6A,D show two examples of poor-quality whole-slide images that contain artifacts. Those images were found in one of the proprietary datasets described in section 2. Figures 6B,E depict selected patches from their corresponding normalized images. We can see that these normalized patches are not projected to a common color space as expected. These results show that the Macenko algorithm does not work in the presence of artifacts in the input image. This is due to the algorithm taking artifact pixels into account while calculating the H&E vectors.

**Figure 7:**
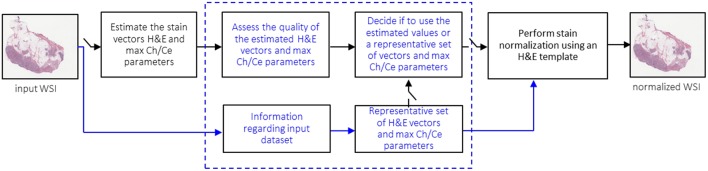
Method to detect and handle problematic H&E estimation (fast_rsn).

A conventional method to identify the presence of artifacts in an image is to divide it into patches, and then analyze each patch in order to detect the presence of artifacts, e.g., by examining the color, intensity, or other features of the image. For example, Vahadane et al. (30) describe a method of dividing the image into patches which are then filtered based on a luminosity threshold for background. Only the remaining patches are used for the estimation of the stain vectors. Such a method however increases the complexity and latency of medical pipelines. We propose a less complex method to detect the cases of problematic H&E estimation for input images. For the rest of the paper, we will refer to this method as fast_rsn. Figure 7 gives an overview of our proposed method.

**Figure 8:**
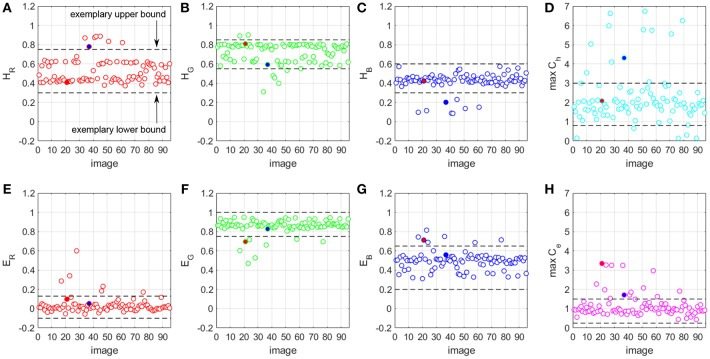
H&E vector components **(A–C,E–G)** and maximum *C*_*h*_ and *C*_*e*_ values **(D,H)** for a prostate biopsy WSI dataset.

For each image in a given dataset, we apply the optimized normalization engine fast_sn to estimate the H&E vectors and the robust maximum (99th percentile) of the pixel stain concentrations *C*_*h*_ and *C*_*e*_. We run fast_sn without the last normalization step, as we only need the estimated H&E vectors and maximum *C*_*h*_ and *C*_*e*_ concentrations. Next, we assess the quality of these values, assuming that poor-quality images have values that deviate from those estimated from good-quality images. By removing images that are assessed to be of poor quality we create a subset of good-quality images. If the image is detected as poor-quality, then the method replaces its estimated parameters with average estimates of the subset of the good-quality images in the same dataset. The image is then normalized using Step 11 in Algorithm 1. Two main components of this proposed method are: (1) detecting the poor-quality images (there is no a-priori knowledge about which images have artifacts and which not), and (2) finding a representative set of H&E and maximum *C*_*h*_ and *C*_*e*_ values. Figure 8 illustrates how the two components work in this paper.

For all images in the dataset, we run the optimized stain normalization engine fast_sn and we extract the estimated H&E components of the three RGB channels (Figures 8A–C,E–G). In addition, we extract the estimated maximum *C*_*h*_ and *C*_*e*_ stain concentration values (Figures 8D,H). We define acceptable ranges for the values of each H&E channel and maximum *C*_*h*_ and *C*_*e*_. Figure 8A shows an example of such a range defined by a lower bound and an upper bound. A value that falls outside its respective range is considered an outlier and any image that has at least one outlier value is considered of poor quality. Otherwise, the image is a good-quality WSI. Once we identify the poor-quality WSIs, we normalize them by using a representative set of H&E vector values and maximum *C*_*h*_ and *C*_*e*_ stain concentration values. This representative set is derived from the values of the good-quality images from the same dataset, by taking the mean of *H*_*R*_, *H*_*G*_, *H*_*B*_, *E*_*R*_, *E*_*G*_, *E*_*B*_, maximum *C*_*h*_ and maximum *C*_*e*_, respectively.

Figures 6C,F show the patches in Figures 6B,E but normalized using the new fast_rsn method. We can already visually notice that fast_rsn projects the images to a more common color space than fast_sn. Moreover, the original images containing the red and blue artifacts (Figures 6A,D) are correctly identified by our new normalization method as poor-quality images as they show up as outliers in Figure 8. For example, Figures 6A,D correspond to the red and blue outliers marked in Figures 8F,H, respectively.

Another variant of the robust normalization method depicted above is the following. Similarly to the fast_rsn method, we first detect the good-quality images and compute the representative set of the H&E and maximum *C*_*h*_ and *C*_*e*_ stain concentration parameters, as described above. Then, instead of replacing the values of the critical parameters only for the outliers (the poor-quality images), we replace them for all images. This method can further reduce the runtime of the stain normalization algorithm and could help in using a more uniform set of stain vectors for normalization across WSIs from the same dataset. We denote this second method by fast_rsn_all.

In our methods we use a set of H&E vectors and maximum *C*_*h*_ and *C*_*e*_ values that are representative of the dataset that contains the identified poor-quality image. This is different from other methods that use color deconvolution with fixed H&E vectors. For example, the main differences when compared to Ruifrok et al. (5) are: (1) our method does not use an arbitrary set of fixed H&E vectors but a set of representative vectors that have been estimated and updated for each dataset and, thus, they are characteristic of the dataset and not agnostic to it, and (2) the set of representative vectors is coming from the same dataset as the input image.

### 3.4. CNN Architecture for Tumor Classification in Whole-Slide Images

To show the benefits of the new stain normalization method described in section 3.3 (fast_rsn) over the stain normalization baseline (fast_sn), we employ a convolutional neural network (CNN) model. We first train a CNN model to detect prostate cancer using images normalized with fast_sn. All images are normalized using their own estimated H&E vectors and maximum *C*_*h*_ and *C*_*e*_ values. We train from scratch the CNN model using images normalized using fast_rsn. Namely, the outliers, i.e., poor-quality images, are normalized using the average of the H&E vectors and maximum *C*_*h*_ and *C*_*e*_ values of the good-quality images. We then compare which model generalizes better on an unseen (test) dataset.

#### 3.4.1. Pre-processing

The size of a WSI at any given resolution level can be too large to process at once. Therefore, we process the WSIs for the tumor detection task in a patch-wise manner. We choose to extract patches from resolution 10X, because 10X contains biological information, such as shape and structure of glands, and arrangement of cells around glands, which are important features for tumor detection. First, we fully normalize the 10X images. We use 70% of the images for training, 15% for validation and 15% for testing. Each patient's biopsies are in a single WSI and this WSI is assigned to exactly one of the train/validation/test partitions to make the testing scenario as realistic as possible (we do not assess the accuracy of our CNN model on data used for training). After normalization, we split the 10X images into patches. The average tumor content in a WSI of our dataset is 5%, thus in order to deal with such a class imbalance, we start extracting patches from the full 10X image using a stride of 512 and reduce it to a stride of 10 when we identify a patch with tumor tissue. The stride is restored to 512 when a patch without tumor region is identified. These stride values ensured a good tumor/non-tumor class balance for our dataset.

#### 3.4.2. CNN Architecture

**Figure 9:**
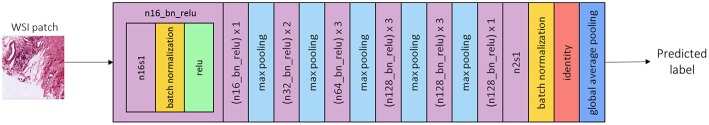
CNN architecture overview. A convolution layer with 16 3 × 3 filters, and a stride of 1 × 1 is denoted by n16s1. The same, followed by a batch normalization layer (yellow) and a ReLU non-linearity (green) is denoted by n16_bn_relu. The learning rate used for training is 0.0001, the optimizer is SGD with momentum 0.9 and the batch size is set to 16.

As an ML model for tumor classification, we use a VGG-inspired CNN architecture that is shown in Figure 9. The model input represents patches extracted as described previously and the output is the predicted label of the input patch, e.g., tumor or non-tumor. The model does not include fully connected layers in order to ensure that the network requires fewer parameters to tune and less GPU memory. All the CNN convolutions have a kernel size of 3 × 3, a stride of 1 × 1 and a ReLU nonlinearity except for the last one. All max pooling layers have a filter size of 2 × 2 and a stride of 2 × 2. For the initialization of weights we used the He normal initializer (31), namely the weights are random but differ in range depending on the size of the previous layer of neurons.

#### 3.4.3. Training and Evaluation

To train the CNN model, we use an SGD optimizer with momentum for optimizing the cross entropy classification loss, a learning rate of 0.0001 and a batch size of 16. After each iteration we save the model weights if the model shows an improvement of the F1 score on the validation dataset. To evaluate the model performance on an unseen test dataset, we use the F1 score and the cross entropy loss.

## 4. Results

We start this section by showing the run-time measurements of our optimized stain normalization algorithm fast_sn for 10X and 40X whole-slide images from the four datasets described in section 2. For 10X images we also show the speedup of fast_sn when compared with a C++ Macenko algorithm implementation based on the MATLAB code available in (20). The latter uses the OpenSlide library (17) for reading the input images. All run-time measurements were collected on a single node with a 10-core Intel® i7-6950X CPU at 3 GHz and 96 GB of RAM. We conclude this section with presenting the CNN accuracy results of fast_sn vs. fast_rsn. For the latter experiments, we used an 8-core Intel(R) Xeon(R) CPU E5-2630 v3, with 64 GB of RAM, and 2 NVIDIA® GTX 1080 TI GPUs.

### 4.1. Optimized Stain Normalization fast_sn: Performance Results

**Figure 10:**
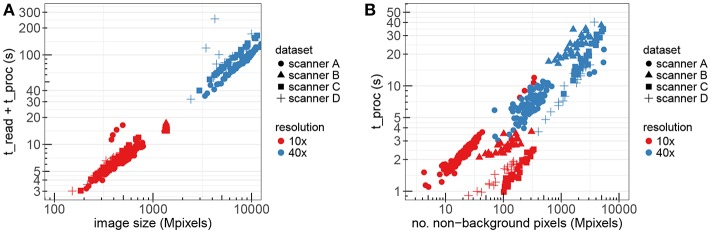
The performance of fast_sn on WSIs from different datasets and resolution factors. **(A)** Read and processing time as a function of the image size. **(B)** Processing time only as a function of non-background pixels.

Figure 10A presents the measurements of the WSI processing time using the optimized stain normalization implementation described in section 3.2. The figure shows the total processing time, including the time to read the images, as a function of the image size in double-logarithmic scale. The different colors correspond to images in 10X and 40X resolutions, while the different markers correspond to the different datasets/scanners. The total number of images used for these experiments was 175.

Figure 10B shows the corresponding processing time only as a function of the number of non-background pixels. We construct a linear regression model where the target variable is t_proc and the dependency between this target variable and the number of non-background nbg pixels can be expressed as t_proc(nbg) = 6.43 · 10^−9^ · nbg + 3.25. This linear model has a multiple *R*-squared metric of 0.7522 which indicates that our implementation scales almost linearly with the number of non-background pixels.

### 4.2. Optimized Stain Normalization *fast_sn* vs. reference: Performance Results

Table 1 reports, for each dataset, the average processing time across all 10X images using fast_sn vs. the average processing time of the reference C++ implementation based on the MATLAB code in (20). As shown in the table, our fast_sn achieves a speedup factor of at least 40 for 10X images, except for the dataset generated with scanner A, where our implementation achieves a speedup of 20. The images in this dataset have a very small percentage of non-background pixels (<5%) which makes the achieved speedup gain less pronounced.

**Table 1:** reference vs. fast_sn: processing time (in seconds) and fast_sn speedup.

**Dataset**	**Proc. time (reference)**	**Proc. time (fast_sn)**	**Proc. speedup**
A	52.4 s	2.5 s	20.7
B	157.4 s	2.7 s	58.1
C	78.3 s	1.5 s	51.8
D	61.7 s	1.4 s	41.8

Table 2 reports for each dataset the average processing and read time across all 10X images using fast_sn vs. reference. As shown in the table, fast_sn achieves a speedup factor of at least 9 for 10X images. This speedup can be further improved by replacing the OpenSlide library used for image loading/storing with an optimized libtiff library. The description of the latter is out of the scope of this paper. The reference implementation could not run for the 40X resolution due to out-of-memory issues, thus we cannot report speedups for 40X images.

**Table 2:** reference vs. fast_sn : processing and read time (in seconds) and fast_sn speedup.

**Dataset**	**Proc.+Read** **time (reference)**	**Proc. + Read** **time (fast_sn)**	**Proc. + Read** **speedup**
A	68.1 s	7.3 s	9.3
B	202.2 s	15.1 s	13.3
C	108.1 s	6.7 s	15.9
D	82.31 s	6.1 s	13.4

### 4.3. Machine Learning Pipeline Accuracy Results: fast_sn vs. fast_rsn

**Figure 11:**
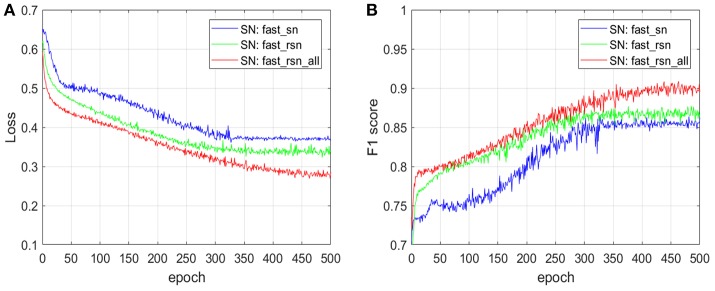
The impact of fast_rsn and fast_rsn_all on the prediction accuracy of an ML pipeline. **(A)** Impact on cross-entropy loss. **(B)** Impact on F1 score.

Figure 11 shows the impact of normalizing the full whole-slide images with the two normalization variants of fast_rsn on the prediction accuracy of the CNN model described in section 3.4, where the input dataset includes images that contain artifacts. We train a convolutional neural network (CNN) architecture with fast_sn-normalized images (SN: fast_sn), with fast_rsn-normalized images (SN: fast_rsn), and with fast_rsn_all-normalized images (SN: fast_rsn_all). We plot the loss (Figure 11A) and F1 score (Figure 11B) on a validation dataset at different training epochs.

The results in Figure 11 show that after the same number of epochs, i.e., 500, training using our new normalization methods described in section 3.3 leads to an improvement in validation loss of up to 30% and in F1 score of up to 5 percentage points. We have also quantified the generalization strength of the CNN models on an unseen (test) dataset. Normalizing the input whole-slide images with fast_rsn improves the F1 score by 5 percentage points (from 0.79 to 0.84). We get similar results when normalizing the input whole-slide images with fast_rsn_all, in which case the F1 score improves by 8 percentage points (from 0.79 to 0.87). Both our normalization methods exhibit a better generalization than fast_sn when tested on an unseen dataset.

## 5. Discussion

In this paper we address two aspects related to the stain normalization pre-processing that is part of modern ML-based pipelines in histopathology.

- Stain normalization can significantly impact the latency of such pipelines especially when dealing with large-size and high-resolution whole-slide images. In this work we have presented a high-performance architecture that enables large-scale processing of high-resolution images.
- Poor-quality images can decrease the decision accuracy of the ML pipelines. We have shown that the stain normalization output can be seriously affected when the input image contains artifacts. We have demonstrated such cases based on a real dataset and proposed an enhanced robust normalization method.

### 5.1. High-Performance Stain Normalization System

Our algorithmic enhancements and system-level optimizations can be applied to other pre-processing algorithms that involve, e.g., automatic detection of background pixels, arithmetic operations such as exponentiation or logarithm, conversions from RGB to optical density domain, pixel sorting and extraction of percentiles, or singular-value decomposition.

Pixel sampling is also a generic optimization that, for example, in the stain normalization algorithm presented in Bejnordi et al. (10) is used indirectly through image tile sampling. Only the pixels in the sampled tiles are used for pixel classification. When using pixel sampling, it is important to define metrics that quantify the impact of using only a part of the available pixels on the quality of the normalization. In our work, we used the Euclidean distance of the H&E vectors and the relative error of the robust maximum of the individual stain concentrations (max *C*_*h*_ and max *C*_*e*_) between the sampling (with different sampling rates) and no-sampling results (14).

Performance-wise, we show that our implementation scales with the amount of tissue present in the image and it processes a 40X whole-slide image in <50 s. This result is comparable with the 60 s scanning time of ultra-fast WSI scanners for 40X images (32). Our high-performance pre-processing system is a first step toward making the stain normalization step suitable for a seamless integration with image scanning in histopathology.

### 5.2. Robust Stain Normalization and Machine Learning

Our proposed method to handle poor-quality images may be applied to other stain normalization algorithms as well. In the case of the stain normalization algorithm under study, the critical parameters that define the normalization quality are the H&E vectors and the robust maximum concentrations of the two stains. In the case of poor-quality images, these parameters are replaced with the average parameter values of the good-quality images from the same dataset. As shown in section 4, such a method can significantly increase the accuracy of ML pipelines. Other color-based normalization algorithms may have similar or other critical parameters. For instance, the Vahadane et al. (30) method also needs to estimate the stain vectors, but it uses a different method based on a dictionary learning-based approach, instead of performing singular-value decomposition.

### 5.3. Other Stain Normalization Methods

Many stain normalization algorithms have been proposed over the past years (5–12). In this paper, we have used the Macenko algorithm (7) for multiple reasons. (1) From a normalization quality perspective, it is one of the best performing algorithms as shown by the in-depth study presented in Zanjani et al. (13). (2) From a system performance perspective, it can be efficiently optimized and parallelized. Building a high-performance and scalable stain-normalization engine is important for the future computer-aided diagnosis systems in histopathology.

Another method that can be used together with stain normalization to improve the variability between the input images and thus increase the accuracy of machine learning pipelines is color augmentation (33). Color augmentation involves various image processing techniques, e.g., random brightness and contrast image perturbations, random shift in hue and saturation, random perturbations in the stain vectors, which are typically manually tuned via visual examination. Lafarge et al. (23) show that color augmentation can improve the ML pipeline accuracy when used alone or in combination with the stain normalization algorithm in Macenko et al. (7). Tellez et al. (34) show that the stain normalization algorithm presented in Bejnordi et al. (10) combined with color augmentation is not necessarily better than color augmentation alone. In this paper, we have focused on optimizing and enhancing the robustness of the stain normalization algorithm.

## 6. Conclusions

We presented a high-performance and scalable system that enables large-scale stain normalization of high-resolution histological whole-slide images. Our pipeline uses a highly-optimized low-level multi-core engine that tackles the memory and runtime bottlenecks of the stain normalization computational load. Moreover, it can be used with different whole-slide image formats, generated by scanners such as Ventana, Aperio, Philips, or Hamamatsu, and it can be easily extended to other whole-slide image formats. Such a system enables the pre-processing of large datasets, which is a critical pre-requisite for any machine learning framework applied to biomedical images.

We also proposed a stain normalization enhancement that improves the accuracy of machine learning pipelines in the presence of poor-quality whole-slide images. To illustrate the robustness of our new normalization method to such images, we employed a machine learning pipeline based on convolutional neural networks that classifies images for detection of prostate cancer. On this exemplary pipeline, our enhanced normalization method increases the F1 score on a test dataset from 0.79 to 0.87.

## Data Availability

The CAMELYON16 and TUPAC16 whole-slide images used for this study can be found here: https://camelyon16.grand-challenge.org/data/ and http://tupac.tue-image.nl/node/3, respectively. These two datasets are publicly available and can be accessed through their corresponding challenge organizers. The other two datasets are owned by hospitals and we don't have permission to make them public.

## Ethics Statement

The data used in this publication has been either publicly available or have been ethically approved for scientific use by the Ethics committee of the University Hospital Zurich.

## Author's Note

This journal article is an extended version of one of our previously published papers (14).

## Author Contributions

AA, MS, SA, and NP conceived the idea, performed the experiments, analyzed the data, and wrote the manuscript. JR and PW provided the proprietary WSI datasets, their corresponding tumor grade annotations and critical medical insights without which the manuscript would not be in its current version. MG and HP contributed to the design of the work, provided managerial support and critical comments.

### Conflict of Interest Statement

AA, MS, SA, NP, MG, and HP are employed by company: IBM Research – Zurich. The remaining authors declare that the research was conducted in the absence of any commercial or financial relationships that could be construed as a potential conflict of interest.

## Footnotes

^1^The content of sections 3.1 and 3.2 of this journal article is partially based on results from our previously published workshop paper (14). This journal paper includes new material as follows: (a) the optimized stain normalization system presented in Stanisavljevic et al. (14) is enhanced with automatic detection of the background pixels threshold; (b) we report the run-time of the optimized system and the speedup gains over the baseline implementation using a larger set of 175 images; (c) we identify a new challenge of the stain normalization algorithm, namely its sensitivity to the quality of the input images, and propose a method to deal with low-quality input images; (d) we evaluate the benefits of the new method over the original normalization algorithm by employing machine learning; (e) we show that our new normalization method increases the classification accuracy of our ML pipeline when compared with the original normalization algorithm.
